# Cirrhosis Progression Is Not Associated with Clinically Significant Alterations in Global Hemostasis Assessed by Thromboelastography

**DOI:** 10.3390/jcm13216614

**Published:** 2024-11-04

**Authors:** Rareș Crăciun, Alina Buliarcă, Daniela Matei, Cristiana Grapă, Iuliana Nenu, Horia Ștefănescu, Tudor Mocan, Bogdan Procopeț, Zeno Spârchez

**Affiliations:** 1 Department of Internal Medicine, “Iuliu Hațieganu” University of Medicine and Pharmacy, 400012 Cluj-Napoca, Romania; craciun.rares.calin@elearn.umfcluj.ro (R.C.); alinasauciuc@yahoo.com (A.B.); bogdan.procopet@umfcluj.ro (B.P.); zsparchez@gmail.com (Z.S.); 2 Gastroenterology Clinic, “Prof. Dr. O. Fodor” Regional Institute of Gastroenterology and Hepatology, 400162 Cluj-Napoca, Romania; horia.stefanescu@irgh.ro (H.Ș.); ioanmocan1@ubbcluj.ro (T.M.); 3 Department of Physiology, “Iuliu Hațieganu” University of Medicine and Pharmacy, 400012 Cluj-Napoca, Romania; iuliana.nenu@gmail.com; 4 UBBMed Department, Babeș-Bolyai University, 400084 Cluj-Napoca, Romania

**Keywords:** coagulation, thromboelastography, viscoelastic tests, liver, cirrhosis, transfusion

## Abstract

(1) Background: Cirrhosis is associated with frequent alterations in standard coagulation tests that do not adequately reflect hemostasis. Thromboelastography provides a global assessment of coagulation and evaluates the functional status of clotting factors, fibrinogen, platelets, and fibrinolysis. The study aimed to assess whether liver disease severity leads to progressive alterations in the thromboelastography-based assessment of coagulation. (2) Methods: Consecutive patients with cirrhosis and abnormal standard coagulation tests (at least one of International Normalized Ratio > 2, platelet count < 50 × 10^3^/µL, fibrinogen < 200 mg/dL) were analyzed using native thromboelastography. (3) Results: A total of 106 patients were included, of whom 69 (65.1%) had a normal thromboelastography. While the standard coagulation tests were significantly worse in patients in the Child C group (*n* = 62, 58.5%) than in patients staged in Child A and B, no significant differences existed between any of the thromboelastography variables. Of the 50 patients (47.1%) with an International Normalized Ratio > 2, only two patients (4%) had features of hypocoagulation, while 26% had features of hypercoagulability on thromboelastography. Patients with a platelet count < 50 × 10^3^/µL had significantly lower platelet function as assessed by thromboelastography, yet only eight patients (20%) met the criteria for platelet transfusion. A thromboelastography-based transfusion protocol might lead to a 94.6% reduction in blood product transfusion indications in a simulation where the included patients would require interventional procedures. (4) Conclusion: Standard coagulation tests showed a poor correlation with thromboelastography. Based on thromboelastography, patients with severe, decompensated liver disease have a preserved hemostasis balance despite abnormal standard coagulation tests. Therefore, standard coagulation tests should not be used to guide the administration of blood products in patients with cirrhosis.

## 1. Introduction

Empirical observations related to the frequency of hemorrhagic episodes in patients with advanced liver disease (variceal and non-variceal gastrointestinal bleeding, various other bleeding episodes in patients with late decompensation), the alteration of standard coagulation parameters (increased prothrombin time (PT), international normalized ratio (INR), thrombocytopenia, and decreased fibrinogen levels), and the premise that a significant proportion of procoagulant factors are synthesized in the liver, have led throughout the 20th century to the deduction that cirrhosis is synonymous with a pro-hemorrhagic status, with the term “spontaneous anticoagulation of the cirrhotic” being frequently used in medical jargon [1]. In the past two decades, research regarding the coagulation status in liver disease patients has dismantled the aforementioned dogma and provided strong evidence suggesting that cirrhotic patients have a “rebalanced” coagulation status: the decline in procoagulant factors is paralleled by commensurate changes in anticoagulant proteins, leading to a novel, albeit more fragile coagulation equilibrium [2,3]. However, standard coagulation tests (SCTs) are not designed to assess the factors associated with rebalanced hemostasis, as they do not consider the compensatory mechanisms involved in the reestablished equilibrium. Thus, SCTs can remain persistently or progressively abnormal throughout liver disease progression but do not provide any actionable information regarding the status of coagulation. For example, endothelial-derived factor VIII and low protein C and S levels contribute to hypercoagulable compensation, which cannot be assessed using SCTs. On the other hand, portal hypertension-related bleeding episodes are mostly associated with an increase in portal pressure beyond the elastic capacity of the containing vessels (Law of Laplace) without a significant contribution from an altered coagulation status [4,5].

Therefore, SCTs appear to be poor indicators for the characterization of bleeding and thrombotic risks in patients with advanced liver disease [6]. Furthermore, guiding therapy based on SCTs may result in unnecessary and potentially life-threatening use of blood products in a futile attempt to correct abnormalities in an already fragile population susceptible to volume overload and various hemodynamic imbalances [7,8]. This observation is supported by clinical evidence from studies that showed that cirrhotic patients carry a risk of deep venous thrombosis and pulmonary embolism [9,10], and their risk of spontaneous or procedure-related bleeding cannot be accurately assessed through standard coagulation tests [8]. A seemingly “rebalanced” coagulation is also reinforced by the fact that the contribution of anticoagulant factors is not considered when exploring thrombin generation in patients with liver disease, which was proven to be much better preserved than initially thought [5]. The endogenous thrombin production test is critical for understanding the hemostatic balance in patients with cirrhosis. However, it seems unlikely to be translated into a clinical tool shortly due to various procedural caveats [9].

Since cirrhotic patients require frequent hospitalizations and a considerable number of minimal or high-risk invasive procedures, rapid point-of-care viscoelastic tests, such as thromboelastography (TEG) and thromboelastometry (ROTEM), have recently emerged as promising techniques for evaluating hemostasis. Compared with SCTs, these methods can provide a global coagulation assessment, assessing each relevant step in clot formation and lysis [10]. Studies using TEG in patients with advanced liver disease have revealed that they tend to maintain balanced hemostasis despite prolonged INR/PT or thrombocytopenia [11,12].

With the TEG method, a whole blood sample is rotated in a cup with a suspended pin. The resistance to rotation is then translated into a graphic figure that provides information concerning clot strength, the dynamics of clot formation, and clot stability. In contrast, SCTs only assess plasmatic events in the hemostatic process, disregarding other whole-blood cellular components and their interactions [12]. TEG records five main parameters: reaction time (R-time), which corresponds to the formation of fibrin; kinetic time (K-time), which records the time from fibrin formation to clot firmness; α-angle, which detects the rate of fibrin formation and is influenced by fibrinogen levels and platelet count; and maximum amplitude (MA), which is dependent on platelet count and function and correlates with clot strength and lysis-30, a parameter that reflects clot dissolution (fibrinolysis) [13]. Based on recent research, TEG can accurately interpret hemostatic changes in patients with cirrhosis, identify and reduce inadequate blood product administration, and improve the management of these patients [10].

Therefore, our study aimed to evaluate the relevance of SCTs in cirrhotic patients compared to a TEG-based assessment. The primary objective was to assess whether liver disease severity was associated with the worsening of the coagulation status assessed by TEG. As a secondary objective, our study evaluated the potential impact of a TEG-based blood product transfusion policy compared to conventional SCT-based policies.

## 2. Materials and Methods

### 2.1. Study Population and Inclusion Criteria

This longitudinal prospective monocentric study was conducted in a high-volume tertiary care hepatology unit from 14 January 2020 to 16 December 2021. A consecutive series of adult patients with cirrhosis and abnormal SCTs were included in this study. The cut-off values for abnormal SCTs were selected according to the Cardiovascular and Interventional Radiological Society of Europe (CIRSE) Clinical Practice Manual, updated in 2021 [14], as follows: platelet count < 50 × 10^3^/mm^3^ and INR > 2. Given the paucity of evidence regarding prophylactic cryoprecipitate transfusions and the heterogeneity of fibrinogen levels predicting bleeding events in patients with cirrhosis, a cut-off level < 200 mg/dL was selected for fibrinogen, which represents the lowest limit of the reference interval in the adult population. The exclusion criteria were age < 18 years, ongoing pregnancy, active or recent history (up to 7 days) of any clinically significant bleeding event (trauma, gastrointestinal bleeding, surgery), recent history (7 days) of blood product transfusion, anticoagulant or antiplatelet therapy, and documented hematological disorders. Patients with hepatocellular carcinoma (HCC) were not excluded from the study population and a subgroup analysis was performed to assess whether HCC has an impact on coagulation. Patients with extrahepatic malignancies were excluded from the study population.

### 2.2. Data Collection and Variables

Baseline characteristics, including demographics, liver disease etiology and staging, and initial laboratory workup, were collected at the time of inclusion. A Sysmex XN-1000 analyzer (Sysmex, Bornbarch, Germany) was used to determine the platelet count, whereas an STA Compact Max 3 analyzer (Stago, Paris, France) was used for standard coagulation assessment and INR.

**Figure 1:**
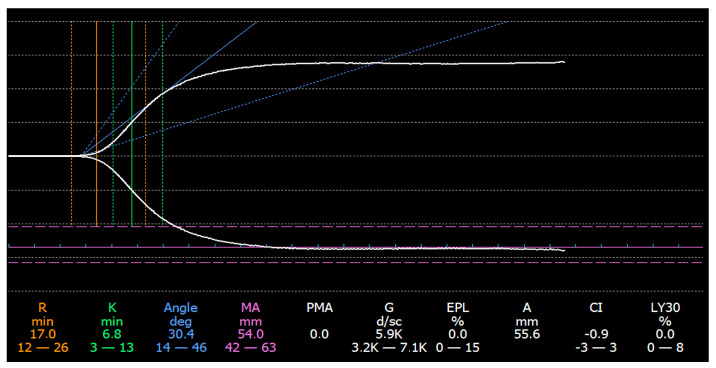
A normal thromboelastography curve. The R-value signifies reaction time, indicating latency to initial fibrin formation. K (kinetics) measures the time to achieve a certain clot strength. The alpha angle assesses fibrin build-up speed in the initiation phase. MA (maximum amplitude) represents ultimate clot strength, dependent on platelets and fibrin. LY30 gauges fibrinolysis. Together, these variables encompass clotting and fibrinolysis processes, providing a comprehensive understanding of clot dynamics and stability in various phases of hemostasis.

Native thromboelastography was performed using a TEG^®^ 5000 Hemostasis Analyzer (Hemoscope Inc., Skokie, IL, USA) point-of-care device. For this purpose, the blood was collected using a double-method polypropylene syringe and light tourniquet pressure. The first 3 mL of blood was discarded from the first syringe and 3 mL was collected in the second syringe. Subsequently, the blood was transferred to a 5 mL polypropylene tube for aspiration and further processing. According to the manufacturer’s recommendations, blood was processed within 4 min from puncture to analysis commencement. Daily maintenance and quality control tests were performed in accordance with the manufacturer’s instructions. A single trained person performed all tests. The TEG results were reported as a standardized curve (Figure 1).

The enzymatic phase, assessing clotting time and the function of the coagulation factors, was assessed using the reaction time (R-time); clot kinetics, depending on the function of fibrinogen, was assessed using the kinetic time (K-time) and the α-angle; platelet function was evaluated using the maximum clot amplitude (MA); and fibrinolysis was assessed using the lysis percentage at 30 min (Ly 30%). The manufacturer provided the reference values for each variable.

### 2.3. Study Design

To address the first primary aim of the study, the patients were divided into two groups based on liver disease severity, as assessed by the Child-Pugh class: Child A + B and Child C. The SCTs and TEG variables were compared between the two groups. A head-to-head correspondence based on the SCTs and TEG variables assessed were drawn as follows: INR and R-time, fibrinogen—K-time and angle, and platelet count—MA).

For the secondary endpoint of the study, a theoretical model was applied, considering each included patient as a potential candidate for an interventional procedure. In this scenario, as all patients had at least one abnormal SCT, they would have been candidates for blood product administration to correct the abnormal SCTs and mitigate periprocedural risks. For this simulation, each instance in which one SCT was abnormal (within the inclusion criteria) was counted (i.e., in the case of a patient with a platelet count < 50,000/mm^3^ and an INR > 2, two instances of blood product use would have been counted). The requirement for blood product use as determined by SCTs was subsequently compared to the in-hospital TEG-based transfusion policy, modeled by the protocol reported by De Pietri L, et al. [15] in which patients would only receive blood products according to their TEG profile (R-time > 40 min, fresh frozen plasma; MA < 30 mm, platelet transfusion; α-angle < 20, cryoprecipitate).

### 2.4. Statistical Analysis

A certified biomedical statistician was used for the statistical analysis utilizing SPSS 29.0.1.0 software (SPSS Inc., Chicago, IL, USA). Continuous variables were described and assessed based on their distribution normality using the Shapiro–Wilk test. Variables exhibiting a normal distribution are presented as mean ± standard deviation (SD) and subjected to comparison using the Student’s *t*-test. Conversely, skewed variables are expressed as median and inter-quartile range (IQR) and compared using the Mann–Whitney U test. Categorical variables were analyzed using the chi-squared test. The established statistical significance threshold was set at *p* < 0.05.

### 2.5. Ethical Considerations

This study was approved by the Ethics Committee of the Host Institution (13541/11.10.2019). The study complied with the updated Declaration of Helsinki and the 2018 Declaration of Istanbul on human subject research. The research objectives were international regulations and primary and secondary legislation. The signing of informed written consent conditioned study participation. Patient data were managed following the European Union’s General Data Protection Regulation.

## 3. Results

### 3.1. Study Population and Baseline Characteristics

The study included a consecutive series of 106 patients with cirrhosis, and their coagulation status was assessed using both SCTs and TEG. The baseline characteristics of the study population are summarized in Table 1.

Regarding the SCTs, *n* = 50 patients (47.1%) had an INR > 2, *n* = 36 (33.9%) had a platelet count < 50 × 10^3^/mm^3^, and *n* = 55 (51.8%) had fibrinogen levels < 200 mg/dL. Overall, 69 (65.1%) patients had normal TEG curves, suggesting no alterations in their hemostatic balance.

### 3.2. Liver Disease Progression and Coagulation

To assess whether liver disease progression was associated with more pronounced alterations in coagulation status, patients were divided into two groups based on liver disease staging assessed using the Child-Pugh class. The first group comprised 44 (41.5%) patients classified as Child-Pugh A and B, and the second group included 62 (58.5%) patients classified as Child-Pugh C. While there were significant differences between the two groups regarding most SCTs (prothrombin time, INR, and fibrinogen levels), there were no differences in any of the TEG-based variables evaluated. Table 2 presents the results.

#### Hepatocellular Carcinoma and Coagulation

On the whole group analysis, there were no statistically significant differences between patients with or without HCC regarding either CCTs or TEG-based variables. A 1:1 propensity-matched analysis included patients staged Child-Pugh A and B, taking into account the MELD-Na score and CCTs, which included 44 patients with HCC. In the setting of a similar conventional coagulation profile, patients with HCC had no significant differences regarding TEG-based variables: R-time 10.62 ± 5.99 vs. 10.92 ± 3.57 min (*p* = 0.84), K-time 5.69 ± 4.38 vs. 6.08 ± 3.30 min (*p* = 0.74), alpha angle 42.11 ± 16.66 vs. 37.28 ± 14.31 (*p* = 0.32), MA 52.94 ± 12.60 vs. 49.05 ± 16.06 (*p* = 0.38), and Ly30 1.61 ± 1.99 vs. 1.48 ± 2.81 (*p* = 0.86), for patients without and with HCC, respectively.

### 3.3. Correspondence Between Standard Coagulation Tests and TEG-Based Variables

To assess whether the SCTs corresponded to TEG-based variables in determining a specific alteration in coagulation dynamics, a head-to-head comparison was performed for each SCT with the TEG-based variable more closely linked to SCT. Consequently, given that INR is primarily a function of coagulation factors, this variable was compared with R-time, which is dependent on both pro-and anticoagulant factors. Platelet count was compared to the maximum clot amplitude, which mainly depends on platelet count and function. Fibrinogen levels were compared to the α angle, which assesses the temporal dynamics of clot formation, a function that is mainly dependent on fibrinogen levels.

#### 3.3.1. INR and R-Time

Almost half of the patients included in the study had an INR of > 2 (*n* = 50, 47.1%). While indeed an INR > 2 was proportionally associated with a longer R-time (9.95 ± 4.31 vs. 12.44 ± 5.55, *p* = 0.01), the mean value for both groups was within the reference interval provided by the manufacturer. Only 2 patients (1.8%) with an INR > 2 had a slightly prolonged R-time, exceeding the reference values, and none met the TEG-based criteria for blood product transfusion. Moreover, 13 (26%) patients with an INR > 2 showed features of hypercoagulability with a shorter R-time.

#### 3.3.2. Platelet Count and Maximum Clot Amplitude

Approximately one-third of the patients had severe thrombocytopenia, with a platelet count of < 50 × 10^3^/mm^3^ (*n* = 36, 33.9%). Thrombocytopenic patients had a lower MA (42.38 ± 13.85 mm vs. 55.02 ± 14.10 mm, *p* < 0.001). The rate of platelet dysfunction assessed using TEG was significantly higher in patients with a low platelet count (20% vs. 2.8, *p* < 0.001). However, of the 36 patients with a platelet count < 50 × 10^3^/mm^3^, only eight (22.2%) met the TEG-based criteria for platelet transfusion.

#### 3.3.3. Fibrinogen Levels and the α-Angle

Hypofibrinogenemia was the most common alteration among the SCTs, as 55 (51.8%) of the patients had fibrinogen levels < 200 mg/dL, and these patients had a lower α angle (42.38° ± 13.85° vs. 55.02° ± 14.10°, *p* < 0.001). Only 4 patients (7.2%) had a α angle below the reference value, and none met the TEG-based criteria for cryoprecipitate transfusion.

#### 3.3.4. Standard Coagulation Tests Versus TEG-Based Blood Product Transfusion Strategies

In the hypothetical scenario, in which the patients included in the study would have needed interventional procedures, all patients had at least one SCT-based criterion requiring correction with blood products (INR > 2, platelet count < 50 × 10^3^/mm^3^, or fibrinogen < 200 mg/dL). Using conventional SCTs, blood product use would have been necessary in 141 cases. In comparison, only eight such instances were supported by a TEG-based transfusion policy, leading to a 94.6% reduction in blood product transfusion indications. A detailed comparison of the two blood-product transfusion strategies is presented in Table 3.

## 4. Discussion

Our results showed a poor correlation between abnormal SCTs and the global assessment of the coagulation profile assessed by TEG, as up to two-thirds of patients with abnormal SCTs had normal TEG curves. Moreover, a significant proportion of patients otherwise deemed hypocoagulable by SCTs show features of hypercoagulation on TEG. While SCTs progressively worsen with liver disease progression because of liver dysfunction and an increase in portal hypertension, the TEG profile remains stable as a result of compensatory mechanisms involved in the rebalanced coagulation that occurs in cirrhosis. Correcting SCT abnormalities with blood products could significantly increase costs and potential adverse events without factual justification. Thus, a TEG-based transfusion may be a more rational approach and could significantly reduce blood product usage.

Viscoelastic hemostatic tests provide real-time dynamic insights into intricate coagulation alterations in cirrhosis. For patients with cirrhosis undergoing invasive procedures or exhibiting active bleeding, TEG and ROTEM should play a role in the initial evaluation, contingent upon the availability of these methods [11,16]. Optimal practice might entail integrating TEG with standard platelet count and fibrinogen testing.

Given our current understanding of coagulation status in patients with cirrhosis, there is a notable interest in coagulation tests that offer a comprehensive perspective on the coagulation system. This has been enforced by the most recent Baveno VII consensus and the European Association for the Study of the Liver Clinical Practice Guidelines on the prevention and management of bleeding and thrombosis in patients with cirrhosis; traditional SCTs such as PT, INR, and activated partial thromboplastin time (aPTT) serve as indicators of general liver dysfunction, but fail to reflect the hemostatic status of patients with advanced liver diseases [17,18]. Although integral to the MELD prognostic score, INR is an inadequate bleeding risk indicator in cirrhosis. Thromboplastin calibration using vitamin K antagonist-treated plasma lacks validation in liver diseases. PT and aPTT assays, which detect only 5% thrombin formation without thrombomodulin, do not reflect activated protein C levels, which are crucial for anticoagulation in cirrhosis [19]. SCTs fail to portray the entirety of in vivo coagulation dysfunction and lack insight into critical factors such as blood flow dynamics, endothelial tissue factors, and platelet function [20,21]. Furthermore, their capacity to assist in the decision-making process regarding plasma or whole-blood administration is limited.

As expected, our study observed a progressive increase in INR, a decrease in fibrinogen levels, and a worsening of thrombocytopenia with liver disease progression, empirically suggesting a more hypocoagulable state. However, noteworthy findings emerged when assessing the TEG parameters. Specifically, the TEG reaction time (r), coagulation time (k), and rate of polymerization, assessed using the alpha angle (α), consistently remained within the normal ranges. While INR is a reliable measure of liver synthetic function, it fails to account for the simultaneous decrease in antithrombotic protein production in the cirrhotic liver. Regarding platelet count, the sheer drop in platelet count does not reflect the activation status of platelets, providing no insights into actual platelet function, and the same rationale can be followed for fibrinogen levels.

For standardization purposes, we decided to use the most frequently utilized SCT cut-offs for impaired coagulation according to the most recent CIRSE recommendations: INR > 2, platelet count < 50 × 10^3^/mm^3^, fibrinogen level < 200 mg/dL [14]. While these values have a significant caveat of not being designed or validated in patients with cirrhosis, they serve as reference points for real-life clinical practice, as many practitioners performing interventional procedures are reluctant to perform even low-risk procedures beyond these cut-offs, despite solid emerging evidence. The fact that only 4% of the patients in our study with an INR > 2 had a corresponding TEG value outside the reference interval and none met the TEG-based criteria for blood product use provides solid evidence that assessing bleeding risks in cirrhosis should not be based on SCTs. Our findings are in line with previously reported data, as a similarly designed and powered study has revealed significant differences in thromboelastography (TEG) parameters between patients in the Child C and Child A + B groups. Although the mean INR and platelet count for all cirrhotic patients were notably outside the normal reference range, the TEG parameters generally fell within normal reference ranges. Specifically, r-time, k-time, and α values in cirrhotic patients increased progressively, while the maximum amplitude values decreased as liver disease severity advanced. However, regression analysis indicated no significant correlations between INR and r-time across any Child-Turcotte-Pugh class (r = 0.01, 0.18, 0.23; *p* = 0.95, 0.39, 0.08, respectively) [22]. These findings are reinforced by stronger evidence from randomized controlled trials. According to a meta-analysis published in 2022, which included data derived from five randomized controlled trials, TEG significantly reduced platelet usage by five times compared to the control group (relative risk: 0.17; 95% CI: 0.03–0.90; *p* = 0.04), but did not significantly affect fresh frozen plasma (FFP) usage. TEG was associated with decreased overall blood product use (*p* < 0.001), including FFP + platelets (*p* < 0.001) and cryoprecipitate (*p* < 0.001). There were no significant differences in bleeding rates or long-term mortality between groups; however, the thromboelastography group exhibited lower 7-day mortality than the control (relative risk [95% CI] = 0.52 [0.30–0.91]; *p* = 0.02) [23]. Another subsequent randomized controlled trial published in 2024 has reached a similar conclusion [24].

Severe thrombocytopenia was observed in almost one-third of the patients. The incidence of platelet dysfunction was notably higher among thrombocytopenic individuals; however, TEG use suggested that only 20% of thrombocytopenic patients require platelet transfusion to rectify the dysfunction. Thrombocytopenia in cirrhosis arises from splenic sequestration, increased platelet destruction, reduced thrombopoietin production, and ethanol-induced toxic effects on platelets [25]. Unless the platelet count is severely low, stable cirrhotic patients may exhibit a pro-thrombotic tendency, with bleeding risks associated more with portal hypertension and collateral vessel formation than with defective hemostasis [24]. Hypercoagulability in cirrhosis is likely due to an elevated factor VIII to protein C ratio and thrombomodulin resistance [26]. These factors, along with an incomplete assessment of the activity of the von Willebrand Factor, cannot be adequately assessed by viscoelastic tests and constitute a major caveat of these methods. Hence, it is a matter of nuance whether TEG, via its MA variable, can assess platelet dysfunction per se, or it is merely a reflection of a low platelet count. However, we decided to use the term platelet dysfunction in relation to MA, as the relationship between MA and thrombocytopenia is non-linear and depends on the interaction between platelets and fibrin via the GP IIb/IIIa axis. In light of the available data, we believe that among all the TEG-based transfusion strategies, platelet transfusion should be approached with the most caution, as the mechanistic relationship regarding platelet activity is less understood. Moreover, another study demonstrated a strong correlation (r = 0.712) between MA-TEG parameters and platelet count, particularly in compensated liver disease. However, this correlation weakened in the Child B and C classes [27]. Thus, we can presume that thrombocytopenia alone cannot entirely account for the decline in MA value between different disease settings, as the correlation with platelet count diminishes with liver disease progression, and the mean platelet count in observed class C cirrhotic patients in the aforementioned study exceeds the accepted cut-off for increased bleeding risk [27].

While TEG has been the most frequently analyzed method in randomized controlled trials for cirrhosis, recent reports also revealed an emerging evidence base for ROTEM. However, at this point, there is insufficient evidence to firmly recommend the use of viscoelastic tests in clinical practice, as the methods still lack adequate validation in relation to a gold standard and still overlook some essential components discussed above [28,29].

Blood transfusions pose inherent risks such as fluid overload, infectious complications, immune reactions, venous thromboembolisms, and antibody sensitization, which are particularly significant for individuals awaiting liver transplantation within this patient population, as these events can significantly alter their prognosis or the changes of a curative treatment [30,31]. Blood management strategies that reduce blood product use have shown positive outcomes in reducing morbidity, mortality, and hospital stay, and implementing low hospital costs. One meta-analysis suggested that integrating thromboelastography can lower blood product use and improve outcomes in patients with cirrhosis [23]. In agreement with this, viscoelastic test-guided therapy significantly decreased transfusion-related adverse events, as evidenced by the meta-analysis conducted by Tang-cheewinsirikul et al. [32]. In our simulation, employing a global coagulation assessment could have prevented over 90% of the potential blood product transfusions. Increased reliance on blood products affects patient clinical outcomes, places financial strain on hospitals, and significantly impacts the entire clinical process, particularly in environments with limited blood donor resources. We acknowledge that the scale of reduction obtained in our modeling can be a matter of debate since our criteria for prophylactic transfusions are arguably loose and rely on recommendations for interventional procedures in general rather than disease-specific guidelines (regarding INR and platelet count) or educated extrapolations from other scenarios (regarding fibrinogen levels). Yet, the thresholds for “standard-of-care” prophylactic transfusions largely resemble the cut-offs used in most of the previously reported literature regarding INR and platelet count [22,30] and, from a clinical practice standpoint, appear to be well ingrained in the perception of most practitioners. Moreover, given the recent understanding of rebalanced coagulation in cirrhosis, it is unlikely that liver-related cut-offs for SCTs will ever emerge. Consequently, while the exact scale of blood product use reduction might vary according to various SCT protocols, the overall benefit is clearly highlighted by the data.

The results of our study have some limitations, beyond limitations regarding the observational design and the relatively small scale of the study, which warrants cautious interpretation. The most important factor is the lack of a gold standard for coagulation assessment, as all viscoelastic tests only analyze the consequences of the entire coagulation process without providing insights into the exact mechanism determining the results. The closest surrogate for a gold standard appears to be the assessment of thrombin generation, which is laborious and beyond the scope of our clinically driven design. The lack of an interventional component also generates another significant caveat. The ideal design to evaluate the role of viscoelastic tests would have been a randomized controlled trial with patients undergoing high-risk interventional procedures while being allocated to two different transfusional policies: TEG- or SCT-based. However, this design has multiple inconveniences. The overall low rate of clinically significant bleeding complications in high-risk procedures and the even lower rate of thrombotic events would require very large sample sizes. Moreover, given the previously discussed evidence on the unreliability of SCTs in cirrhosis, allocating patients to the SCT-based transfusion policy group poses multiple ethical concerns. Nevertheless, our study provides solid clinical data regarding the promise of TEG as a reliable point-of-care tool to assess the hemostatic profile of patients with cirrhosis and provide sufficient rationale to limit unjustified blood product use in clinical practice.

## 5. Conclusions

Thromboelastography revealed that the rebalanced hemostatic profile of patients with cirrhosis appears to be preserved throughout liver disease progression, despite progressive alterations in SCTs. Using viscoelastic tests to evaluate the need for blood product use in the frequent cases of interventional procedures in these patients could significantly contribute to reducing product use, related costs, and adverse events. The nuanced assessment of how the hemostatic balance shifts, oscillating between procoagulant and anticoagulant states, necessitates an individualized approach by the clinician, and viscoelastic tests offer a potential solution for elucidating this intricate puzzle.

## Figures and Tables

**Table 1:** The baseline characteristics of the study population.

Variable	Value
Age (years)	58.12 ± 11.24
Liver disease etiology (*n*, %)	
Alcohol-related liver disease	52 (49.05%)
Hepatitis B	16 (15.09%)
Hepatitis C	23 (21.69%)
Other	15 (14.15%)
Liver disease staging (*n*, %)	
Child-Pugh A	21 (19.8%)
Child-Pugh B	23 (21.7%)
Child-Pugh C	62 (58.5%)
Albumin (g/dL)	2.93 ± 0.68
Total bilirubin (mg/dL)	2.9 (1.47–7)
International Normalized Ratio	1.88 (1.44–2.78)
Platelet count	59.5 (41.75–100.75)
Fibrinogen (mg/dL)	198.5 (137.25–257.75)

Frequencies were reported in brute number and percentage; variables with a normal distribution were reported as mean ± standard deviation; variables with a non-parametric distribution were reported as median (interquartile range).

**Table 2:** Comparison of standard coagulation tests and TEG-based variables according to liver disease severity.

	Child-Pugh A + B *n* = 44 (41.5%)	Child-Pugh C *n* = 62 (58.5%)	*p*-Value
**Standard coagulation tests**			
International Normalized Ratio	1.43 (1.25–1.61)	2.57 (1.77–3.37)	<0.001
Prothrombin time (sec)	22.1 (18.6–25.6)	35.65 (21.25–50.05)	<0.001
Platelet count (×1000/mm^3^)	54 (37–71)	75 (41–109)	0.16
Fibrinogen (mg/dL)	248 (189.5–306.5)	155 (90.5–210.5)	<0.001
**TEG-based variables**			
R-time (min)	10.78 ± 5.09	11.40 ± 5.08	0.51
α-angle (°)	40.13 ± 15.76	41.33 ± 16.50	0.70
K-time (min)	5.1 (2.75–7.45)	4.3 (2.3–6.3)	0.28
Maximum amplitude (mm)	51.35 ± 14.53	50.29 ± 15.75	0.72
Ly30 (%)	0.4 (0.2–0.5)	0.3 (0.2–0.5)	0.74

Variables with a normal distribution were reported as mean ± standard deviation; variables with a non-parametric distribution were reported as median (interquartile range).

**Table 3:** Comparison between standard coagulation tests and TEG-based blood product transfusion strategies.

Blood Product Type	Standard Coagulation Tests	TEG-Based Variables	*p*-Value
Fresh frozen plasma/coagulation factors	INR > 2 50 instances	R-time > 40 min 0 instances	<0.001
Platelet transfusion	Platelet count < 50 × 10^3^/mm^3^ 36 instances	MA < 30 mm Eight instances	<0.001
Cryoprecipitate	Fibrinogen levels < 200 mg/dl 55 instances	α-angle < 20° 0 instances	<0.001
Total count	141 instances	Eight instances	<0.001

INR—international normalized ratio; MA—maximum clot amplitude.

## Data Availability

The raw data supporting the conclusions of this article will be made available by the authors on request, given that the dataset is part of an ongoing research project.
